# Targeting oxidized phosphatidylcholines in SOD1-associated ALS: therapeutic potential of PC-OxPL-VecTab^®^

**DOI:** 10.3389/fnins.2025.1620181

**Published:** 2025-07-08

**Authors:** Andreia Gomes-Duarte, Sofia Pascoal, Rob Haselberg, Marina Sogorb-Gonzalez, Sander van Deventer

**Affiliations:** VectorY Therapeutics, Matrix Innovation Center VI, Amsterdam, Netherlands

**Keywords:** amyotrophic lateral sclerosis, superoxide dismutase 1, oxidized phosphatidylcholines, gene therapy, adeno-associated viruses

## Abstract

Amyotrophic lateral sclerosis (ALS) is a devastating neurodegenerative disease characterized by progressive motor neuron degeneration. Mutations in the superoxide dismutase 1 (SOD1) gene account for a significant fraction of familial ALS (fALS) cases. Oxidative stress and oxidized phosphatidylcholines (PC-OxPL) contribute to neuroinflammation and neuronal damage, and to motor neuron degeneration in ALS. We previously demonstrated the therapeutic efficacy of an AAV-delivered anti-PC-OxPL single-chain variable fragment (PC-OxPL-VecTab^®^) in neutralizing PC-OxPL toxicity in the periphery and central nervous system (CNS), but the therapeutic potential of PC-OxPL-VecTab^®^ has not been investigated in the context of fALS and SOD1-associated ALS. We report that PC-OxPL accumulation contributes to the pathological phenotypes associated with SOD1^G93A^ iPSC-derived motor neurons and the corresponding mouse model. The current findings further demonstrate that PC-OxPL-VecTab^®^ is efficacious in neutralizing the downstream effects of SOD1-associated PC-OxPL accumulation, such as altered gene expression and axonal health in SOD1 motor neurons, as well as a pathological lipid profile in the SOD1^G93A^ mouse model. Collectively, the present study underscores the significance of PC-OxPL dysfunction in the context of SOD1 genotypes and sheds light on the potential of PC-OxPL-VecTab® for therapeutically targeting ALS.

## 1 Introduction

Amyotrophic lateral sclerosis (ALS) is a devastating neurodegenerative disorder marked by the progressive degeneration of motor neurons, leading to premature death. Mutations in the SOD1 gene account for 10–20% of familial ALS (fALS) cases (Ingre et al., 2015). Mutant SOD1 is associated with the misfolding and aggregation of the SOD1 protein (Hayashi et al., 2016). This SOD1 toxic gain-of-function is believed to induce neuronal cell death through mechanisms such as excitotoxicity, calcium overload, oxidative stress, and mitochondrial dysfunction, ultimately contributing to the onset and progression of ALS (Hayashi et al., 2016; Kawamata and Manfredi, 2010; Peggion et al., 2022).

Metabolic dysfunctions in SOD1^G93A^ mice have been associated with increased oxidative stress and lipid peroxidation (Ravera et al., 2018). These mechanisms can result in the formation of oxidized phosphatidylcholines (PC-OxPL), which are not merely byproducts of oxidation but actively engage in various biological processes (Itri et al., 2014). PC-OxPLs exert their biological activity through multiple pathways, influencing processes such as plasma membrane integrity, apoptosis, and immune response regulation (reviewed in Dong and Yong, 2022). In fact, they have been found in multiple sclerosis (MS), frontotemporal lobe dementia (FTD), Parkinson’s disease (PD), and Alzheimer’s disease (AD) tissues, and are implicated in chronic neuroinflammation, neuronal damage, and cell death (Dong and Yong, 2022). In a recent study, PC-OxPL were identified as key effectors in the pathophysiological processes associated with sporadic ALS (sALS), including significant transcriptomic changes in ALS-associated genes, marked TDP-43 pathology, and motor neuron death (Gomes-Duarte et al., 2025).

PC-OxPL neoepitopes are specifically recognized by the monoclonal antibody E06 (Binder et al., 2016). Interestingly, transgenic mice expressing E06 in a single-chain variant format (scFv) have been shown to display ameliorated disease phenotypes related to asthma, atherosclerosis, liver disease, and other indications (Que et al., 2018; Sun et al., 2020; Tsimikas and Witztum, 2024). We developed an optimized AAV-delivered anti-PC-OxPL single-chain variable fragment (PC-OxPL-VecTab®), based on E06 scFv, which effectively neutralized PC-OxPL toxicity in the CNS (Gomes-Duarte et al., 2025). However, its therapeutic potential in addressing SOD1-associated ALS pathology remains unexplored.

The present study investigates the contribution of PC-OxPL to the pathology associated with SOD1^G93A^ iPSC-derived motor neurons and a corresponding mouse model, as well as the potential for PC-OxPL-VecTab^®^ as a novel therapeutic approach for SOD1-associated ALS.

## 2 Materials and methods

### 2.1 In vitro

#### 2.1.1 Culturing of wild-type and ALS motor neurons

Wild-type and ALS (SOD1^G93G^ and ^G93A^) iCell motor neuron lines were generated from a healthy donor using CRISPR gene editing to introduce ALS-SOD1 mutations (FCDI, Madison, WI). Seeding and maintenance of iCell motor neurons were performed as earlier described in (Gomes-Duarte et al., 2025), with minor modifications. Wt SOD1^G93G^ and ALS SOD1^G93A^ iCell motor neurons were cultured in a volume of 500 or 100 μL on a 12- (3,513, Corning) or 96-well plate (165,305, Thermo Fisher Scientific)/OMEGA^NMJ^ device (eNUVIO), respectively. Medium changes were performed every 2 to 3 days by replacing 50–75% of the culture media, as recommended by the provider. The OMEGA^NMJ^ device (eNUVIO) was used according to the manufacturer’s instructions to specifically assess axonal phenotypes. Wt and SOD1^G93A^ motor neurons were initially seeded in chamber #1 to stimulate axonal projection toward chamber #2 through the microchannels.

#### 2.1.2 AAV transduction

Generation of the AAV5.2-PC-OxPL-VecTab^®^ is described in detail in Gomes-Duarte et al. (2025). In the present study, the AAV5.2-PC-OxPL-VecTab^®^, which encompasses the mouse E06 transgene, was exclusively used. Human iPSC-derived cultures were transduced at a multiplicity of infection (MOI) of 5×10^6^ in culturing medium at 7 days post-seeding.

#### 2.1.3 Phosphocholine (PC) treatment

*1-palmitoyl-2-(9-oxononanayl)-phosphocholine* (PONPC) (870605P-1MG, Avanti Polar Lipids) and *1-hexadecanoyl-2-octadecanoyl-sn-glycero-3-phosphocholine* (PSPC) (850456P-25MG, Avanti Polar Lipids) solutions were prepared as described previously (Gomes-Duarte et al., 2025). iCell motor neurons cultured in the OMEGA^NMJ^ microfluidic device were treated with 25 μM of PONPC (PC-OxPL) or PSPC (non-oxidized control) for 24 h at 7 days post-transduction to maximize axonal damage.

#### 2.1.4 Gene expression studies and functional enrichment analysis

Neuronal culture preparation, RNA and DNA extraction, nCounter Gene expression analysis using NanoString panels (neuropathology and neuroinflammation), and associated Gene Ontology (GO) studies have been performed as described in (Gomes-Duarte et al., 2025). Data mining was performed using ALS- (Abel et al., 2012; D’Erchia et al., 2017) and ferroptosis-related databases (Zhou and Bao, 2020). Gene Ontology (GO), or functional enrichment, analysis was performed using g: Profiler (version e112_eg59_p19_25aa4782), with g: GOSt (functional profiling) (Kolberg et al., 2023). A g: SCS significance threshold of 0.05 was used as the method for multiple testing correction. Functional enrichment was calculated as the -log_10_ of the adjusted *p*-value (−log_10_(padj)).

#### 2.1.5 Immunocytochemistry and analysis

PC-OxPL detection on *in vitro* using wt and SOD1^G93A^ motor neurons was performed as described previously (Gomes-Duarte et al., 2025). Analyses were carried out using off-the-shelf quantification methods from the CellReporterXpress Software. PC-OxPL profile was defined as the percentage of positive cells (PC-OxPL+), as quantified by the cell scoring protocol. Axonal damage was assessed after 24 h of PONPC exposure by incubating permeabilized cultures present in the OMEGA^NMJ^ microfluidic device with the neuronal marker class III *β*-tubulin (TuJ1), as described previously (Gomes-Duarte et al., 2025). For downstream analyses, imaging of the OMEGA^NMJ^ distal compartment next to the microgrooves was conducted by the ImageXpress Pico system at a 20x magnification. Distal axons were segregated, and the number of axons projected toward the muscle compartment was numerically quantified as a marker for axonal damage.

### 2.2 In vivo

#### 2.2.1 Intrathecal delivery and tissue collection in SOD1^G93A^ mice

Transgenic SOD1^G93A^ mice (Raju et al., 1996) were acquired from Jackson Laboratory (JAX Stock #002726, hybrid C57BL/6 and SJL background). This line carries a high transgene copy number (also known as G1H). Animal experiments were performed at the Charles River Laboratory facility in Kuopio (Finland). Five-week-old (45 days) male SOD1^G93A^ mice were injected intrathecally with AAV5.2-PC-OxPL-VecTab® or vehicle (n = 12 mice/group). Naïve male wt mice of the same age were used as controls (n = 12). Due to gender effects in disease progression and survival, male mice were chosen for this study to reduce variability and group sizes (Heiman-Patterson et al., 2005). Animals were anesthetized in induction chambers (2.0–5.0% isoflurane, in 70% N2O and 30% O2; flow 600 mL/min) and fixed in a prone position on the surgery platform on the heating pad. The heating pad was set to 37°C, and the rectal probe was inserted into the animal with the help of a lubricant. Anesthesia was maintained at 1.5–2.5%. After skin incision and dura mater visualization, a canula attached to a Hamilton syringe mounted on a microinfusion system was then inserted into the subarachnoid space. Five μL of AAV5.2-PC-OxPL-VecTab^®^ (1.25×10^12^ genome copies (gc)/mouse) or vehicle solution (PBS/Pluronic 0.001%) were injected at a rate of 0.5 μL/min. On the scheduled aging day (day 45, 70, or 90), the animals were euthanized by deep anesthesia with pentobarbital (180 mg/kg, i.p.). Terminal blood was collected via cardiac puncture, and 200 μL of plasma was separated for downstream biomarker analysis. Animals were transcardially perfused with ice-cold heparinized (Heparin 2.5 IU/mL) saline. Six wt/vehicle-SOD1^G93A^ mice and five AAV5.2-PC-OxPL-VecTabs^®^-treated SOD1^G93A^ were blindly selected for bioanalysis and perfused with saline. From these mice, tissue samples were collected from the cervical spinal cord, lumbar spinal cord, brain cortex, and liver and snap-frozen in liquid nitrogen before storing at −80°C for bioanalysis.

#### 2.2.2 Biodistribution analysis of vector DNA and PC-OxPL-VecTab^®^ mRNA

DNA isolation from liver, spinal cord, and brain tissues was performed using the DNeasy blood and tissue kit (QIAGEN). TaqMan primers and probes specific for the vector DNA promoter sequence were used to measure the vector gc by TaqMan qPCR (Thermo Fisher Scientific). The amount of vector DNA was calculated based on a plasmid standard curve, and the results were reported as gc/μg genomic DNA. RNA isolation from liver, spinal cord, and brain tissues was performed using the RNeasy Mini kit QIAGEN, according to the manufacturer’s protocol, and the samples were eluted in 30 μL of RNase-free water. RNA concentrations were determined by NanoDrop ONEC (Thermo Scientific). cDNA synthesis by reverse-transcription PCR was performed with the Maxima First Strand cDNA Synthesis Kit for RT-qPCR (Thermo Scientific). qPCR amplification was performed with customized TaqMan primers for PC-OxPL-VecTab® expression and with TaqMan primers for *Mus musculus* hypoxanthine phosphoribosyltransferase 1 (*Hprt1*) housekeeping gene (Mm03024075_m1, Thermo Fisher Scientific). The transgene mRNA expression levels were calculated as fold-change to *Hprt1* expression.

#### 2.2.3 LC–MS quantitation of selected PC-OxPL species

A total of 50 μL of plasma was extracted from six wt/SOD1^G93A^ mice and five AAV5.2-PC-OxPL-VecTab^®^-treated mice according to Folch’s protocol (Folch et al., 1957). The supernatants were evaporated to dryness and resolved with methanol. The resolved samples were analyzed using an Agilent 1,290 HPLC system with binary pump, multisampler, and column thermostat with a Zorbax Eclipse plus C-18, 50 × 2.1 mm, 1.8 μm; 40°C column using a gradient solvent system consisting of mobile phase A: acetonitrile/water 60:40, 5 mM NH4Ac, 0.05% formic acid and mobile phase B: 2-propanol/acetonitrile 90:10, 5 mM NH4Ac, 0.05% formic acid. The gradient consisted of 32–97% B in 21 min, stop time after 27 min. The flow rate was set at 0.4 mL/min, and the injection volume was 5 μL. The HPLC was coupled with an Agilent 6470 Triple Quadrupole mass spectrometer (Agilent Technologies, Santa Clara, USA) with electrospray ionization source parameters optimized. Analysis was performed with multiple reaction monitoring, according to Solati et al. (2021). The identification of the analytes was performed by the characteristic mass transition and retention time. For quantification, external POVPC and SOVPC standards (Cayman Chemical, Ann Arbor, MI) were used.

### 2.3 Statistical analysis

Statistical analysis was performed with GraphPad Prism (version 10.2.3). Differences in PC-OxPL as measured by immunohistochemistry, as well as differences in specific PC-OxPL levels between groups measured at a specific timepoint (day 45, 70, or 90) were evaluated using an unpaired two-tailed Student’s t-test. No statistical predictions were used to determine sample size (n), but the n used resembled what is generally performed in the field. For *in vivo* analysis (LC–MS), outliers were removed using the ROUT method (Q = 1%) (Motulsky and Brown, 2006), taking the full dataset per treatment group into account (time dimension neglected). Comparisons between untreated SOD1 and SOD1-treated mice were made using a two-way ANOVA, encompassing 70 and 90 days together, and taking SOD1 as the normalized (average = 100%) dataset. The difference was expressed as log_2_fold-change (log_2_FC). A full two-way ANOVA model was fitted using Bonferroni correction for multiple comparisons.

All values are expressed as mean ± the standard deviation (SD) or standard error of the mean (SEM). Statistical significance was considered for *p*-values (p) ≤ 0.05, *p* < 0.0001: ****; *p* < 0.001: ***; *p* < 0.01: **; *p* < 0.05: *; *p* ≥ 0.05: ns or not significant.

## 3 Results

### 3.1 SOD1^G93A^ motor neurons accumulate PC-OxPL and exhibit pathological phenotypes

**Figure 1:**
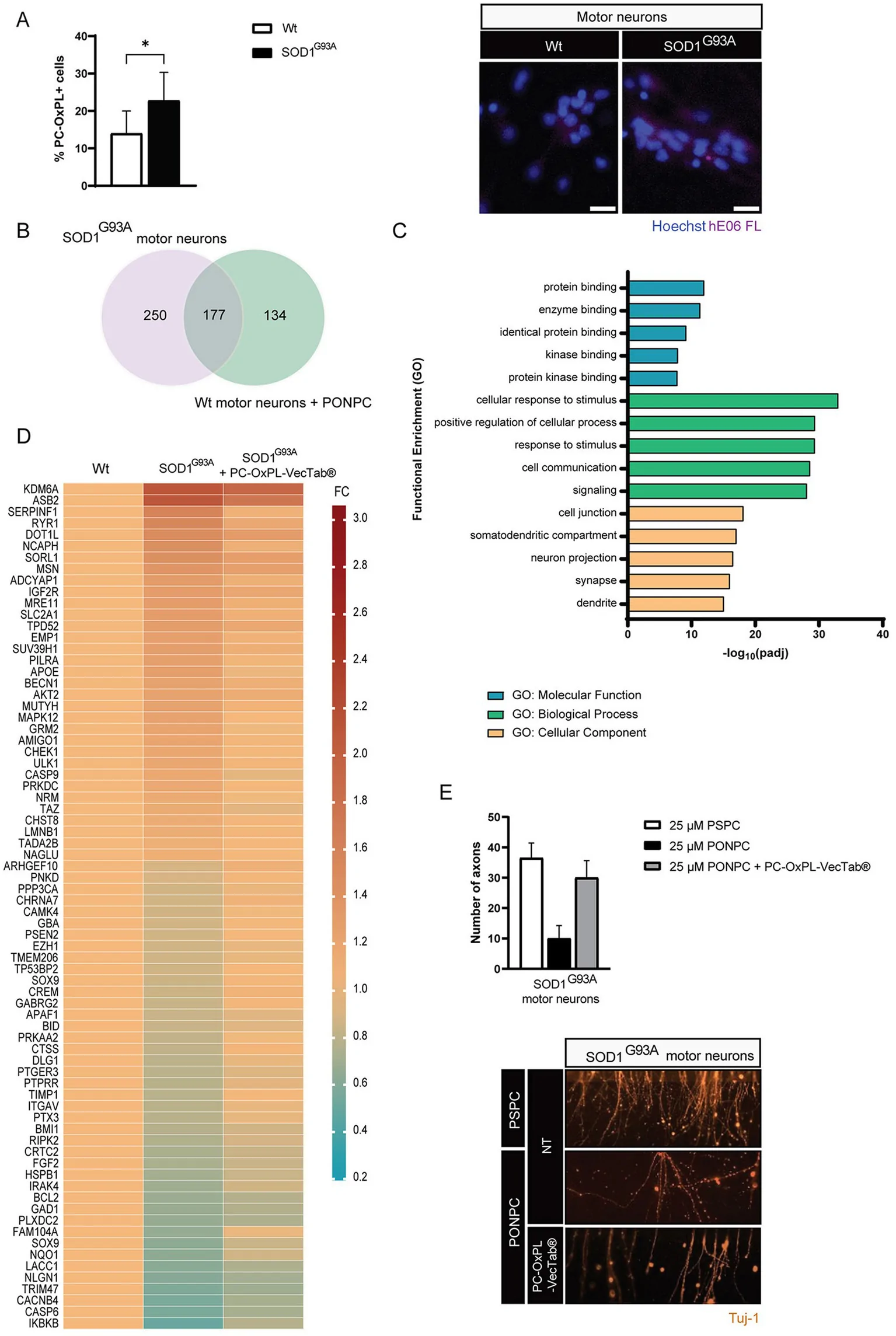
**(A)** *Left panel:* PC-OxPL expression in SOD1^G93A^ motor neurons (% of PC-OxPL+ cells). Values are expressed as means (Wt = 13.97; SOD1^G93A^ = 22.77) ± SD. Unpaired two-tailed *t*-test; *p = 0.0283. *n* = 7–8 replicates. *Right panel:* Representative images showing increased % of PC-OxPL+ cells in SOD1^G93A^ in comparison to Wt motor neurons. Scale bar = 20 μM (PC-OxPL, Cy5). **(B)** Venn diagram depicting overlapping DE transcripts between SOD1^G93A^ and wt motor neurons exposed to PC-OxPL (PONPC). Both NanoString neuropathology and neuroinflammation gene expression panels were considered in the analysis. **(C)** Functional enrichment analysis of transcriptomic changes following AAV5.2-PC-OxPL-VecTab^®^ transduction of SOD1^G93A^ motor neurons. Only the five most enriched terms of each category are graphically represented (GO, Gene Ontology; padj, adjusted *p*-value; MF, molecular function; BP, biological process; CC, cellular component). **(D)** Heatmap representation of SOD1^G93A^ transcriptome normalization following AAV5.2-PC-OxPL-VecTab^®.^ Cell values represent the mean FC in relation to wt levels (FC, fold-change). **(E)** Axonal pathology in SOD1^G93A^ motor neurons exposed to PC-OxPL is mitigated by PC-OxPL-VecTab®. *Upper panel:* Values are expressed as means ± SD and normalized to the 25 μM PSPC condition. The number of axons was counted on the distal compartment as per TuJ-1 detection, used as a neuronal marker; *n* = 2 independent experiments. *Lower panel:* Representative panel of the distal compartment depicting PC-OxPL toxicity and neutralization by PC-OxPL-VecTab^®^ in SOD1^G93A^ motor neurons.

To characterize the general PC-OxPL profile in ALS (SOD1^G93A^) motor neurons, we used the hE06 antibody and immunofluorescence. This approach revealed a modest yet significant accumulation (20%) of PC-OxPL in SOD1^G93A^ motor neurons compared to their wt counterparts, as evidenced by an elevated proportion of PC-OxPL+ motor neurons (Figure 1A). NanoString analysis was performed to identify relevant gene expression signatures in SOD1^G93A^ motor neurons. Approximately 30% of the transcriptome analyzed was altered in SOD1^G93A^ motor neurons in comparison to wt motor neurons across neuropathology (NP) and neuroinflammation (NI) panels (NP = 257/770; NI = 210/770) (Supplementary Table S1). Of the differently expressed (DE) transcripts across panels, the slight majority were upregulated (57% compared to 43% downregulated) because of the SOD1^G93A^ genetic background (Supplementary Table S1). Remarkably, a 40% overlap was found on DE transcripts resulting from the SOD1^G93A^ mutation and previously identified signatures of motor neuron exposure to PC-OxPL (*n* = 177/427) (Gomes-Duarte et al., 2025; Figure 1B; Supplementary Table S1). Most of the altered genes (approximately 70%) were associated with neuropathological markers, particularly those related to neurological disease pathways, axonal and dendrite morphology, as well as neurotransmitter release, response, reuptake, and vesicle trafficking. The most DE up- and downregulated transcripts in SOD1^G93A^ motor neurons are listed in Table 1.

**Table 1:** List of the most DE genes in SOD1^G93A^ in comparison to wt motor neurons.

Gene name	Description	FC	Wt + PC-OxPL overlap (Gomes-Duarte et al., 2025)
CCND1	Cyclin D1	2.54	Yes
SMC1A	Structural maintenance of chromosome 1A	2.27	Yes
HGF	Hepatocyte growth factor	2.19	No
KDM6A	Lysine demethylase 6A	2.11	Yes
ASB2	Ankyrin repeat and SOCS Box containing 2	2.10	Yes
SLC17A6	Solute carrier family 17 member 6	1.92	No
PLA2G16	Phospholipase A2 group XVI	1.80	No
NTS	Neurotensin	1.77	Yes
DBH	Dopamine beta-hydroxylase	1.68	No
CD8A	CD8alpha	1.64	No
CACNB4	Calcium voltage-gated channel auxiliary subunit beta 4	0.57	Yes
CASP6	Caspase 6	0.57	Yes
JUN	Transcription factor Jun	0.57	No
MEF2C	Myocyte enhancer factor 2C	0.56	Yes
FRMPD4	FERM and PDZ domain containing 4	0.54	Yes
COL4A1	Collagen type IV alpha 1 chain	0.51	Yes
EPHA5	EPH receptor A5	0.50	No
INPP5F	Inositol polyphosphate-5-phosphatase F	0.42	No
CALB1	Calbindin 1	0.40	Yes
FN1	Fibronectin 1	0.15	No

### 3.2 PC-OxPL-VecTab^®^ normalizes SOD1-associated transcriptome and axonal pathology

The efficacy of PC-OxPL-VecTab^®^ in neutralizing ALS-related *in vitro* and *in vivo* phenotypes has been described previously (Gomes-Duarte et al., 2025). Functional enrichment analysis using g: Profiler was used to determine the most significantly enriched pathways associated with PC-OxPL-VecTab® expression in SOD1^G93A^ motor neurons. The five most significantly enriched terms in each GO category were investigated in more detail for SOD1^G93A^ motor neurons transduced with AAV5.2-PC-OxPL-VecTab^®^ by comparison to SOD1^G93A^ motor neurons (Figure 1C). The most representative molecular pathways associated with AAV5.2-PC-OxPL-VecTab^®^ transduction included protein binding, particularly kinases. Biological processes activated by AAV5.2-PC-OxPL-VecTab^®^ transduction included a general response to stimulus, cell communication, and signaling. Finally, most of the transcripts changed in response to the AAV5.2-PC-OxPL-VecTab^®^ transduction were associated with neuronal projections, including the synaptic and dendritic cellular compartments. The complete functional enrichment analysis retrieved from g: GOSt can be found in Supplementary Table S2. A closer analysis of gene expression changes was performed to evaluate the efficacy potential of PC-OxPL-VecTab^®^ in normalizing SOD1-associated gene expression. Of the total DE genes between SOD1^G93A^ and wt motor neurons, 20% (*n* = 74/435) were restored toward wt levels following AAV5.2-PC-OxPL-VecTab^®^ transduction (Figure 1D; Supplementary Table S3). Of note, four of the transcripts displaying the most altered expression in SOD1^G93A^ were found within the normalized transcriptome set (IKBKB, CASP6, KDM6A, and ASB2). Ferroptosis is an iron-dependent form of cell death that has been found to contribute to selective motor neuron death in ALS (Wang et al., 2022, 2024a). SOD1 mutations (e.g., H46R or G85R) can trigger the formation of amyloid fibrils which promote ferroptosis through mitochondrial damage and oxidative stress (Wang et al., 2024b). Interestingly, several ferroptosis hits (Zhou and Bao, 2020) were found among the altered genes in SOD1^G93A^ motor neurons and subsequently normalized by PC-OxPL-VecTab^®^ (Figure 1D; Supplementary Table S4). Next, we investigated the ability of PC-OxPL-VecTab^®^ to prevent axonal damage, a hallmark of degenerating neurons in ALS (Fischer and Glass, 2007), using an OMEGA^NMJ^ microfluidic device by comparing axonal damage in PC-OxPL-exposed SOD1^G93A^ motor neurons in the presence or absence of PC-OxPL-VecTab^®^. Axonal damage, as measured by the number of affected axons, caused by the SOD1^G93A^ mutation in combination with motor neuron exposure to PC-OxPL, was almost completely prevented in the presence of PC-OxPL-VecTab^®^ (Figure 1E).

### 3.3 Plasma PC-OxPL levels are lowered upon intrathecal PC-OxPL-VecTab^®^ administration in SOD1^G93A^ mice

**Figure 2:**
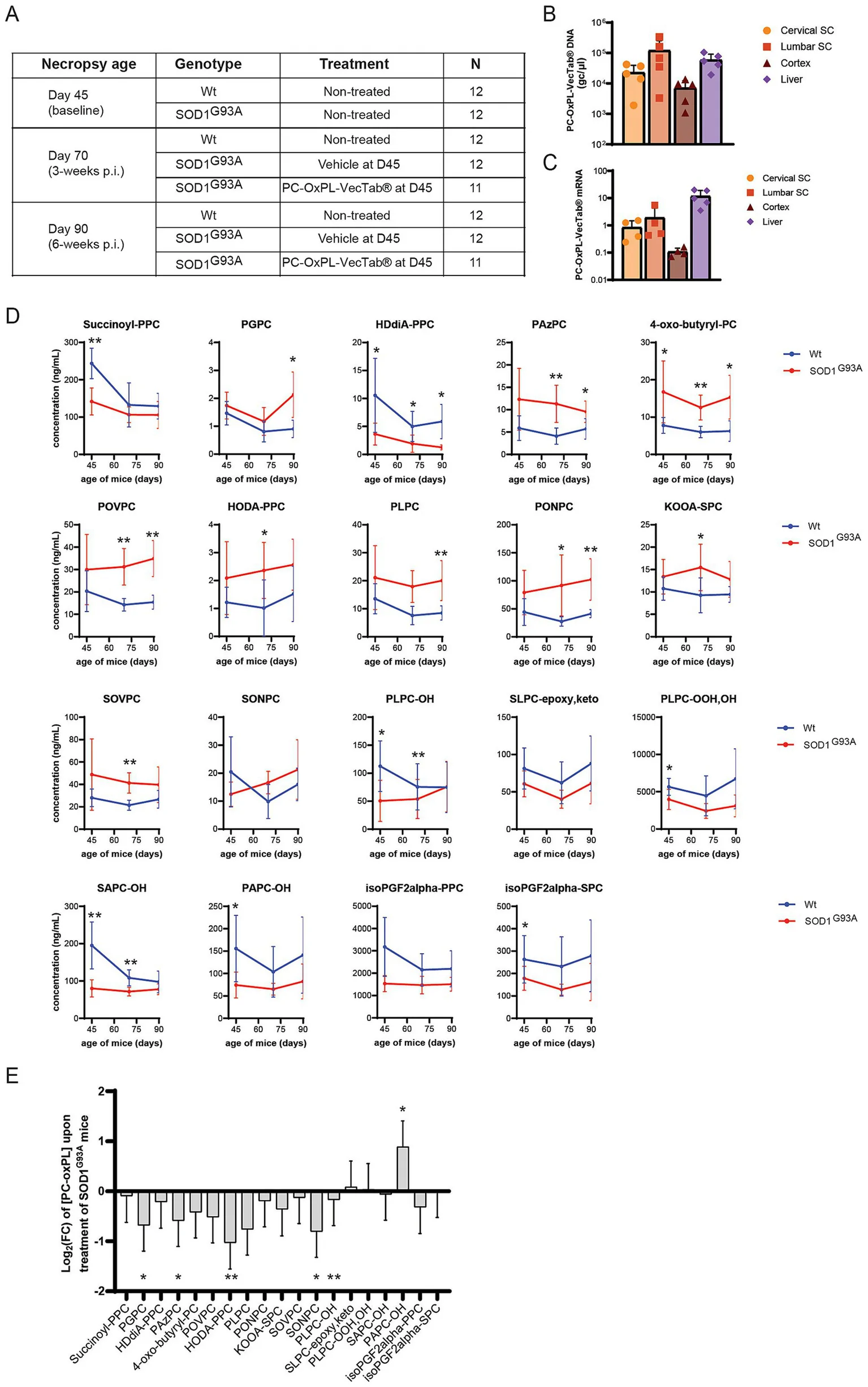
**(A)** Study design and treatment groups in wt and SOD1^G93A^ mice. **(B)** Vector DNA levels (gc/μg DNA) in cervical and lumbar spinal cord, brain cortex, and liver in PC-OxPL-VecTab^®^-treated SOD1^G93A^ at day 70 of age (3 weeks post-injection). vDNA levels were determined by qPCR analysis and quantified based on a plasmid standard curve. Columns represent mean ± SD. **(C)** PC-OxPL-VecTab^®^ mRNA expression levels in cervical and lumbar spinal cord, brain cortex, and liver in PC-OxPL-VecTab^®^-treated SOD1^G93A^ at day 70 of age (3 weeks post-injection). Transgene mRNA levels were measured by RT-qPCR and quantified as FC to the housekeeping (HKG) gene mHPRT1. Columns represent mean ± SD. One of the animals with low levels of vDNA (< 3×10^3^ gc/μg DNA) showed undetectable levels of PC-OxPL-VecTab^®^ mRNA. **(D)** Plasma PC-OxPL concentrations (in ng/mL) determined in wt and SOD1^G93A^ mice over time (killed at days 45, 70, and 90 of age). Values are expressed as means ± SD. Unpaired t-test with Welch correction; ****p < 0.0001; ***p < 0.001; **p < 0.01; *p < 0.05, *n* = 4–6 depending on lipid and time point. **(E)** Log_2_(FC) converted the effect of PC-OxPL-VecTab^®^-treated SOD1^G93A^ mice vs. vehicle-SOD1^G93A^ mice for the 19 detected PC-OxPL species. A negative value represents a decrease in a specific PC-OxPL upon treatment with AAV5.2-PC-OxPL-VecTab^®^. Two-way ANOVA; ****p < 0.0001; ***p < 0.001; **p < 0.01; *p < 0.05. *n* = 9–12, depending on lipid and treatment.

To study the efficacy of PC-OxPL-VecTab^®^ *in vivo*, a SOD1^G93A^ mouse model was used, in which primary pathology consists of motor neuron degeneration in the spinal cord and brain cortex, followed by progressive limb paralysis (Gurney et al., 1994). The study synopsis is shown in Figure 2A. Oxidized lipids, including PC-OxPL species, have been previously identified in this mouse model (Tu et al., 2023).

To achieve sufficient transduction and expression in the relevant CNS areas, lumbar intrathecal infusion was selected as the route of administration for the AAV5.2-PC-OxPL-VecTab^®^ in mice. The transduction and expression levels of the AAV-PC-OxPL-VecTab^®^ in the cervical and lumbar spinal cord, brain cortex, and liver of SOD1^G93A^ mice were determined by qPCR in five mice from the treatment group euthanized at day 70 of age. Lumbar IT injection resulted in high levels of vDNA transduction in the lumbar region of the spinal cord, followed by the cervical spinal cord, brain cortex, and liver (Figure 2B). A similar biodistribution profile was observed for the PC-OxPL-VecTab^®^ mRNA expression levels, with lumbar spinal cord and liver expressing the highest levels and cortex the lowest (Figure 2C).

To evaluate PC-OxPL levels in wt and in SOD1^G93A^ mice, a targeted LC–MS assay was used (Solati et al., 2021). Plasma samples obtained from wt and SOD1^G93A^ mice killed at days 45, 70, and 90 were analyzed. In these samples, 19 PC-OxPL species were identified and quantified out of 22 included. Apparent PC-OxPL differences were observed between SOD1^G93A^ vs. wt mice, with about half of the PC-OxPL species being higher and the other half being lower in concentration (Figure 2D). For most species, no prominent time trends were observed, and in individual cases, the differences were significant, as indicated.

Next, the effect of scFv treatment was evaluated by determining plasma PC-OxPL levels in SOD1^G93A^ mice treated with PC-OxPL-VecTab^®^. The mice were killed at 25 and 45 days post-injection, and PC-OxPL species were quantified in these samples. No significant difference was observed in PC-OxPL levels on day 70 vs. day 90. Therefore, the data were pooled and compared to the same (pooled) dataset from untreated SOD1^G93A^ mice (Supplementary Table S5). Overall, 16 out of 19 PC-OxPL species were decreased upon treatment (Figure 2E; Supplementary Table S5). Specifically, three PC-OxPL species were statistically significantly decreased by PC-OxPL-VecTab^®^ treatment (HODA-PPC, SONPC, and PAPC-OH). The overall treatment effect was considered highly significant (*p* < 0.001).

## 4 Discussion

PC-OxPL neurotoxicity is increasingly linked to CNS diseases, including ALS (Dong and Yong, 2022; Gomes-Duarte et al., 2025). This study aimed to investigate whether PC-OxPL neurotoxicity extends to SOD1-associated ALS and if it can be mitigated using an AAV-delivered anti-PC-OxPL scFv, PC-OxPL-VecTab^®^. Our findings demonstrate that in both iPSC-derived motor neurons and a mouse model, the SOD1^G93A^ background exhibits a pathological transcriptome and elevated PC-OxPL levels, which are partially corrected by PC-OxPL-VecTab^®^ treatment.

Transcriptomic profiles of SOD1^G93A^ and wt motor neurons exposed to PC-OxPL were remarkably similar. Most of the overlapping genes were associated with neuronal morphology, neuroplasticity, and disease pathology, suggesting that the oxidative environment caused by PC-OxPL induces neuronal dysfunction beyond general inflammation. Oxidative stress in SOD1-associated ALS (Itri et al., 2014; Peggion et al., 2022) likely contributes to PC-OxPL generation, as evidenced by the increased PC-OxPL profile in these neurons.

Despite extensive validation of PC-OxPL targeting using E06, the underlying mechanisms are not fully elucidated, but two main processes have been proposed: (i) masking of PC-OxPL, inhibiting their biological effects and/or (ii) elimination from the biological system by enzymatic activity or excretion (Upchurch et al., 2022). Our findings support these mechanisms by showing the (i) normalization of a PC-OxPL-associated pathological phenotype in motor neurons and (ii) decreased PC-OxPL following PC-OxPL-VecTab^®^ expression in a SOD1^G93A^ mouse model. Kinase-related enzymatic activity was prominent following the PC-OxPL-VecTab^®^ transduction of motor neurons. Kinases regulate various cellular processes, primarily through the phosphorylation of specific protein substrates (Pang et al., 2022). Notably, phosphorylated SOD1 has been found to co-deposit with other ALS-related proteins, such as TDP-43, in the motor neurons of ALS patients (Trist et al., 2022). It is tempting to speculate that PC-OxPL accumulation triggers or enhances TDP-43 and SOD1 proteinopathy in ALS by initiating molecular events related to kinase activity, and that those can be mitigated in the presence of PC-OxPL-VecTab^®^. Concerning primary processes associated with PC-OxPL-VecTab^®^ expression in SOD1^G93A^ motor neurons, those were mainly linked to cell-to-cell communication and neuronal projections, specifically synapses and dendrites. These observations align with the effect of PC-OxPL-VecTab^®^ in preventing axonal damage induced by the PC-OxPL treatment of SOD1^G93A^ motor neurons and with the expected target engagement mechanism for PC-OxPL-VecTab^®^.

The effect size of PC-OxPL targeting in SOD1^G93A^ mice upon PC-OxPL-VecTab^®^ treatment was smaller than that reported previously (Upchurch et al., 2022) for a similar, yet peripheral treatment approach. This may reflect the IT administration route, which permits peripheral leakage while still predominantly targeting CNS regions. The current study focused on the plasma-derived PC-OxPL lipidome. In the CNS, toxic fatty acids produced in hyperactive neurons are transferred to astrocytes by apoE-lipid particles, where they are processed through mitochondrial *β*-oxidation (Ioannou et al., 2019). OxPL, in particular, has been described to be recognized by TREM2 receptors and neutralized by microglia (Dong et al., 2021). In contrast, outside the CNS, oxidized lipids are likely to be stored and metabolized in hepatocytes, thus preventing the accumulation of toxic lipid species in cell membranes (Takahashi et al., 2015). Together, these findings suggest that the PC-OxPL-VecTab^®^ effectiveness profile may be compartmental or even ALS-type specific. Emerging perspectives propose that PC-OxPL generation may not be a random process, and that different species may exhibit different biological functions (Dong and Yong, 2022).

Despite the novelty of our findings, there are some limitations to acknowledge. First, the effect of PC-OxPL-VecTab^®^ *in vivo* does not provide clarity on the specificity or affinity of this approach to the different PC-OxPL species. Distinct lipid and PC-OxPL profiles may emerge in sALS, given the differing mechanisms compared to those involved in SOD1-associated ALS or even other forms of fALS (Mejzini et al., 2019). Future LC–MS-based pull-down studies could clarify the target engagement potential of PC-OxPL-VecTab^®^ in ALS. Second, the *wt* mice group does not control for the potential effects of the intrathecal injection in the readouts since *wt* mice were not infused with vehicle as the SOD1^G93A^ mice were. Third, PC-OxPL levels should ideally be measured in CSF to study the treatment effect. However, collected CSF volumes were less than the required assay input (~10 vs. 50 μL, respectively), and plasma was, therefore, selected as a substitute biofluid. Finally, while functional efficacy of PC-OxPL-VecTab^®^ has been shown before in a different model (Gomes-Duarte et al., 2025), the efforts designed to determine improvements in motor function and survival in the SOD1^G93A^ mouse model will further improve confidence in PC-OxPL-VecTab^®^ from a therapeutic standpoint.

Over the past years, several therapeutic interventions targeting oxidative stress in ALS have shown moderate but encouraging results, including delayed neurodegeneration and disease progression (Park and Yang, 2021). It remains unclear whether PC-OxPL neutralization is sufficient to fully cover SOD1-associated neuropathology. SOD1 is primarily degraded through the ubiquitin-proteasome system (UPS), and subunits of this complex are susceptible to oxidative damage (Manton and Chandra, 2014), which may contribute to the accumulation of misfolded SOD1 and disease severity. One hypothesis is that treatment with PC-OxPL-VecTab^®^ creates a favorable intracellular environment, facilitating the clearance of existing toxic SOD1 aggregates by motor neurons through canonical degradation mechanisms. In turn, this may aid motor neuron survival and slow disease progression (Tsekrekou et al., 2024). Future studies should shed light on whether targeting PC-OxPL is sufficient to consider improvements on clinical endpoints for SOD1-associated ALS, also by comparison to current approved strategies, such as Tofersen^®^ (Hamad et al., 2025). PC-OxPL-VecTab^®^ may also complement other strategies targeting ALS, such as antisense or antibody-based therapies, or be considered for other indications.

To conclude, this study presents the first evidence of PC-OxPL accumulation and toxicity in SOD1-associated ALS. We show that the neutralization of PC-OxPL using AAV delivery is a strategy that may be therapeutically relevant and applicable to fALS forms or other pathologies. These findings underscore the potential of PC-OxPL-VecTab^®^ for targeting ALS by neutralizing PC-OxPL-related neurotoxicity.

## Data Availability

The datasets presented in this study can be found in online repositories. The names of the repository/repositories and accession number(s) can be found at: https://www.ncbi.nlm.nih.gov/geo/, GSE281698; https://www.ncbi.nlm.nih.gov/geo/, GSE281714.

## Glossary

AAV: Adeno-associated virus
AD: Alzheimer’s disease
ALS: Amyotrophic lateral sclerosis
BSA: Bovine serum albumin
CNS: Central nervous system
CSF: Cerebrospinal fluid
DE: Differently expressed
ERAD: Endoplasmic reticulum-associated degradation
EtOH: Ethanol
fALS: Familial amyotrophic lateral sclerosis
FC: Fold-change
FDR: False discovery rate
FTD: Frontotemporal dementia
FOV: Field of view
gc: Genome copies
GO: Gene Ontology
iPSC: Induced pluripotent stem cells
E06: OxPL-targeting antibody
LC–MS: Liquid chromatography – mass spectrometry
MOI: Multiplicity of infection
mRNA: Messenger RNA
NAFLD: Non-alcoholic fatty liver disease
NT: Non-transduced
OxPL: Oxidized phospholipids
PC-OxPL: Oxidized phosphatidylcholine
PBS: Phosphate-buffered saline
PC: Phosphatidylcholine
PD: Parkinson’s disease
PDL: Poly-D-lysine
PONPC: *1-palmitoyl-2-(9-oxononanayl)-phosphocholine*
PSPC: *1-hexadecanoyl-2-octadecanoyl-sn-glycero-3-phosphocholine*
PUFA: Polyunsaturated fatty acids
QC: Quality control
ROS: Reactive oxygen species
rRNA: Ribosomal RNA
RT: Room temperature
sALS: Sporadic amyotrophic lateral sclerosis
sc: spinal cord
scFv: Single-chain variable fragment
SD: Standard deviation
SEM: Standard error of the mean
SOD1: Superoxide dismutase 1
TuJ1: Class III *β*-tubulin
TBS: Tris-buffered saline
vDNA: Vector DNA
Wt: Wild type
