# Correction: Targeting oxidized phosphatidylcholines in SOD1-associated ALS: therapeutic potential of PC-OxPL-VecTab^®^

**DOI:** 10.3389/fnins.2026.1960544

**Published:** 2026-08-26

**Authors:** Andreia Gomes-Duarte, Sofia Pascoal, Rob Haselberg, Marina Sogorb-Gonzalez, Sander van Deventer

**Affiliations:** VectorY Therapeutics, Matrix Innovation Center VI, Amsterdam, Netherlands

**Keywords:** amyotrophic lateral sclerosis, superoxide dismutase 1, oxidized phosphatidylcholines, gene therapy, adeno-associated viruses

There was an error in Figure 1A as published. During revision, Figure 1A was updated in response to a reviewer request to display absolute, non-normalized values rather than values normalized to the Wt group. An error occurred during this update, and the published figure does not reflect the intended values.

**Figure 1:**
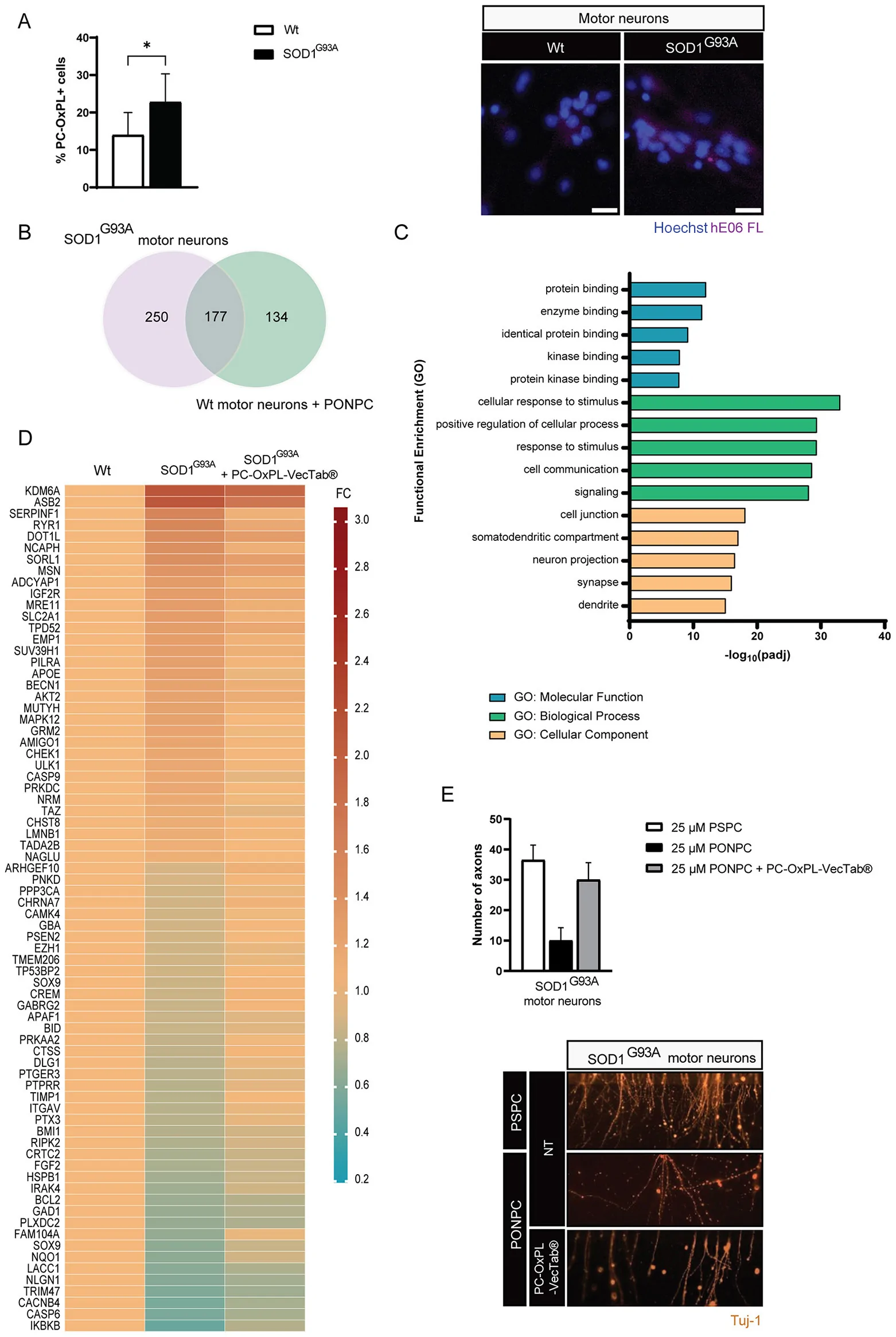
**(A)** *Left panel*: PC-OxPL expression in SOD1^G93A^ motor neurons (% of PC-OxPL+ cells). Values are expressed as means (Wt = 13.97; SOD1^G93A^ = 22.77) ± SD. Unpaired two-tailed *t*-test; ^*^p = 0.0283. *n* = 7–8 replicates. *Right panel*: Representative images showing increased % of PC-OxPL+ cells in SOD1^G93A^ in comparison to Wt motor neurons. Scale bar = 20 μm (PC-OxPL, Cy5). **(B)** Venn diagram depicting overlapping DE transcripts between SOD1^G93A^ and wt motor neurons exposed to PC-OxPL (PONPC). Both NanoString neuropathology and neuroinflammation gene expression panels were considered in the analysis. **(C)** Functional enrichment analysis of transcriptomic changes following AAV5.2-PC-OxPL-VecTab^®^ transduction of SOD1^G93A^ motor neurons. Only the five most enriched terms of each category are graphically represented (GO, Gene Ontology; padj, adjusted *p*-value; MF, molecular function; BP, biological process; CC, cellular component). **(D)** Heatmap representation of SOD1^G93A^ transcriptome normalization following AAV5.2-PC-OxPL-VecTab^®^. Cell values represent the mean FC in relation to wt levels (FC, fold-change). **(E)** Axonal pathology in SOD1^G93A^ motor neurons exposed to PC-OxPL is mitigated by PC-OxPL-VecTab^®^. Upper panel: Values are expressed as means ± SD and normalized to the 25 μm PSPC condition. The number of axons was counted on the distal compartment as per TuJ-1 detection, used as a neuronal marker; *n* = 2 independent experiments. Lower panel: Representative panel of the distal compartment depicting PC-OxPL toxicity and neutralization by PC-OxPL-VecTab^®^ in SOD1^G93A^ motor neurons.

The corrected Figure 1A appears below.

Consequently, there was also an error in the caption of Figure 1A. The corrected caption of Figure 1A appears below.

The corrected figure results in an updated statistical value. The direction of the effect, interpretation of the data, and conclusions of the study remain unchanged.

The original version of this article has been updated.

